# ChatGPT, medical research and scientific writing

**DOI:** 10.51866/lte.545

**Published:** 2024-01-06

**Authors:** Somsri Wiwanitkit, Viroj Wiwanitkit

**Affiliations:** 1 PhD, Private Academic and Ediorial Consultant, Bangkok Thailand. Email: somsriwiwan@hotmail.com; 2 MD, FRFM, Adjunct professor, Center for global health research, Saveetha medical college, Saveetha Institute of medical and technical sciences, India.

**Keywords:** ChatGPT, Medical, Research, Writing

**Dear editor,**

We read with great interest the article titled ‘Use of ChatGPT in medical research and scientific writing’.^1^ The introduction of ChatGPT, an artificial intelligence (AI) language model based on the GPT-3.5 architecture, has resulted in major advances in scientific writing and medical research. ChatGPT is being used by researchers for a variety of tasks, including conducting automated literature reviews, generating structured outlines and providing drafting/editing assistance. The tool’s capacity to adapt language for varied audiences, handle citations, assist collaborative writing and peer review and create tables and figures has substantially enhanced efficiency in these disciplines.

Despite the advantages of ChatGPT, issues have been raised about ethical problems, prejudice, accuracy and originality. When employing ChatGPT, users must ensure transparent data sourcing and validation because the tool supplements human efforts but cannot replace critical thinking. Researchers must uphold their integrity and guarantee that Al-assisted content adheres to research ethics. Furthermore, as advised by the International Committee of Medical Journal Editors, noting the use of AI in papers increases accountability.

As AI language models advance, ethical controls and restrictions must be implemented to avoid the dissemination of damaging ideas and incorrect information. If there is insufficient human monitoring or verification, a chatbot may provide a fake reference, which could lead to additional problems.^2,3^ For success, new tactics and a range of training choices are required, considering the risks associated with relying solely on a large data source. Given the potentially negative or unanticipated repercussions, using chatbots raises ethical concerns.

Moving forward, it is critical to solve the challenges associated with the use of ChatGPT and improve the tool’s capabilities. Researchers should concentrate their efforts on creating strategies to reduce biases and increase the accuracy of AI-generated material. This could entail improving the training process by combining diverse and representative datasets and adopting effective validation approaches. Efforts should also be taken to guarantee that ChatGPT retains its originality and does not generate plagiarised content. Furthermore, researchers should continue to emphasise the value of critical thinking and human expertise when using AI tools such as ChatGPT. It is critical to view ChatGPT as a tool that supplements rather than replaces researchers’ judgement and subject knowledge. The transformative promise of ChatGPT can be achieved by fostering a symbiotic interaction between researchers and AI tools, driving scientific progress while preserving ethical standards.
